# Unravelling the role of intratumoral bacteria in digestive system cancers: current insights and future perspectives

**DOI:** 10.1186/s12967-024-05320-6

**Published:** 2024-06-07

**Authors:** Weiqin Lu, Aihemaitijiang Aihaiti, Paziliya Abudukeranmu, Yajun Liu, Huihui Gao

**Affiliations:** 1 General Surgery, Cancer Center, Department of Vascular Surgery, Zhejiang Provincial People’s Hospital (Affiliated People’s Hospital), Hangzhou Medical College, Hangzhou, Zhejiang China; 2 Aksu First People’s Hospital, Xinjiang, China; 3 Cancer Center, Department of Hospital Infection Management and Preventive Medicine, Zhejiang Provincial People’s Hospital (Affiliated People’s Hospital), Hangzhou Medical College, Hangzhou, Zhejiang China

**Keywords:** Intratumoral bacteria, Digestive system cancers, Functions, Mechanisms, Therapeutic strategy

## Abstract

Recently, research on the human microbiome, especially concerning the bacteria within the digestive system, has substantially advanced. This exploration has unveiled a complex interplay between microbiota and health, particularly in the context of disease. Evidence suggests that the gut microbiome plays vital roles in digestion, immunity and the synthesis of vitamins and neurotransmitters, highlighting its significance in maintaining overall health. Conversely, disruptions in these microbial communities, termed dysbiosis, have been linked to the pathogenesis of various diseases, including digestive system cancers. These bacteria can influence cancer progression through mechanisms such as DNA damage, modulation of the tumour microenvironment, and effects on the host’s immune response. Changes in the composition and function within the tumours can also impact inflammation, immune response and cancer therapy effectiveness. These findings offer promising avenues for the clinical application of intratumoral bacteria for digestive system cancer treatment, including the potential use of microbial markers for early cancer detection, prognostication and the development of microbiome-targeted therapies to enhance treatment outcomes. This review aims to provide a comprehensive overview of the pivotal roles played by gut microbiome bacteria in the development of digestive system cancers. Additionally, we delve into the specific contributions of intratumoral bacteria to digestive system cancer development, elucidating potential mechanisms and clinical implications. Ultimately, this review underscores the intricate interplay between intratumoral bacteria and digestive system cancers, underscoring the pivotal role of microbiome research in transforming diagnostic, prognostic and therapeutic paradigms for digestive system cancers.

**Supplementary Information:**

The online version contains supplementary material available at 10.1186/s12967-024-05320-6.

## Introduction

Digestive system cancers pose a significant global health challenge due to their high incidence and mortality rates [1–5]. Epidemiological analyses demonstrated that digestive system cancers, encompassing malignancies of the oesophagus, stomach, liver, pancreas, colon and rectum, significantly contribute to the overall health burden worldwide [6–8]. Recent advancements in endoscopic techniques and minimally invasive surgery have greatly improved the outcomes and quality of life for individuals diagnosed with early-stage digestive system cancers [9–13]. Simultaneously, the advent of personalised medicine, facilitated by molecular profiling and targeted therapies, has revolutionised the landscape of digestive system cancer management [14–16]. Notably, immunotherapeutic interventions, particularly effective for treating mismatch repair-deficient colorectal cancer and hepatocellular carcinoma, offer promising prospects for enhancing patient outcomes [17–20]. Despite these advances in detection and treatment modalities, managing digestive system cancers continues to encounter challenges [21, 22]. Early detection remains a formidable obstacle, particularly for pancreatic and oesophageal cancers, which often manifest symptoms at advanced stages [23–25]. Variability in survival rates across different digestive system cancers reflects the inherent biological diversity, tumour heterogeneity and varied treatment responses, highlighting the complexity of these malignancies [26–29]. As research progresses, the potential of intratumoral bacteria in digestive system cancer development is increasingly recognised, paving the way for innovative approaches to digestive system cancer therapy (Table 1) [30–32].

**Table 1:** The profiling and functions of intratumoral bacteria in digestive system cancers

Cancer type	Bacteria and other pathogens	Expression	Role	Underlying mechanisms	Functions	Publication year	Reference
HCC	LPS	increased	carcinogenesis	LPS/TLR4/NF-κB/hepatomitogen epiregulin/caspase-3	promote proliferation and prevention of apoptosis.	2012	[90]
HCC	LPS	increased	carcinogenesis	DSS/LPS	promote hepatic tumorigenesis, inflammation, and fibrosis	2016	[60]
HCC	LPS	increased	carcinogenesis	LPS/TLR4/NF-Κb/ROS	promote hepatic inflammation and proliferation	2010	[88]
HCC	LPS	increased	carcinogenesis	LPS/TLR4/NF-Κb/Snail	promote cell epithelial-mesenchymal transition, and tumour invasion and survival	2012	[78]
HCC	DCA	increased	carcinogenesis	gut bacteria/DCA/IL-1β/IL-6/CXCL1	provokes SASP phenotype in HSCs, and facilitates HCC development	2013	[74]
HCC	GLCA	increased	carcinogenesis	GLCA/CXCL16/CXCR6/INF-γ	promote antitumor immunosurveillance	2018	[75]
HCC	SCFA	increased	carcinogenesis	fibre-fermenting bacteria/inulin/SCFA	promote bile acid dysmetabolism, early onset of cholestasis, hepatocyte death, neutrophilic inflammation, and icteric HCC	2018	[76]
HCC	butyrate-producing genera, and producing-lipopolysaccharide genera	a decrease in butyrate-producing genera, and an increase in producing-lipopolysaccharide genera	carcinogenesis	/	/	2019	[61]
HCC	Escherichia coli, Shigella, Faecalibacterium, Ruminococcus, and Ruminoclostridium	a decrease in Escherichia coli and Shigella, and an increase in Faecalibacterium, Ruminococcus, Ruminoclostridium	carcinogenesis	/	promote amino acid and glucose metabolism	2019	[87]
HCC	Bifidobacterium, Bacteroides, and Ruminococcaceae	a decrease in Bifidobacterium, and an increase in Bacteroides and Ruminococcaceae	carcinogenesis	/	promote systemic inflammation	2019	[62]
CRC	Fusobacterium nucleatum	increased	carcinogenesis	/	/	2012	[98]
CRC	Fusobacterium nucleatum	increased	carcinogenesis	Gal-GalNAc/Fap2	promote CRC Metastases	2016	[101]
CRC	Fusobacterium nucleatum	increased	carcinogenesis	Fap2/E-cadherin/β-catenin	promote inflammatory responses and CRC cell proliferation	2016	[102]
CRC	Fusobacterium nucleatum	increased	carcinogenesis	Fap2/TIGIT	inhibit immune cell activity	2015	[103]
CRC	Fusobacterium nucleatum	increased	carcinogenesis	TLR4/MYD88/NFκB/miR21/RASA1/MAPK	promote CRC cell proliferation and invasion, and tumour growth	2017	[79]
CRC	EPEC	increased	carcinogenesis	EspF/MMR	promote spontaneous mutation frequency in host cells, and increase host cell ROS levels	2013	[106]
CRC	ETBF	increased	carcinogenesis	BFT/STAT3/NF-κB/IL-17R	promote pro-tumoural immature myeloid cell recruitment and tumour formation	2018	[71]
GC	Bacterial diversity	decreased	carcinogenesis	/	/	2018	[141]
GC	Bacterial diversity	decreased	carcinogenesis	/	/	2014	[140]
GC	Bacterial diversity	decreased	carcinogenesis	/	/	2018	[139]
GC	Bacterial diversity	increased	carcinogenesis	/	/	2018	[142]
GC	Bacterial composition and diversity	decreased	carcinogenesis	/	/	2019	[145]
PC	Bacterial composition and diversity	increased	carcinogenesis	TLR/PD-1	promote immune tolerance and tumour formation	2018	[77]
PC	Gammaproteobacteria	increased	carcinogenesis	CDD_L_	promote drug resistance	2017	[72]
PC	Pseudoxanthomonas, Saccharopolyspora and Streptomyces	increased	anti-carcinogenesis	/	promote antitumor immunity	2019	[63]
PC	3-IAA	increased	anti-carcinogenesis	myeloperoxidase/3-IAA/glutathione peroxidase 3/glutathione peroxidase 7/ROS	impair PC cell metabolic fitness, and proliferation	2023	[157]
PC	somatic-cell-associated bacteria	increased	carcinogenesis	cell-type-specific gene expression and pathway	regulate cell motility and immune signalling	2022	[158]
PC	Fusobacterium nucleatum	increased	carcinogenesis	GM-CSF, CXCL1, IL-8, and MIP-3α	promote cell proliferation, migration, and invasion	2023	[159]
EC	Bacterial diversity	decreased	anti-carcinogenesis	/	/	2017	[163]
EC	Fusobacterium nucleatum	increased	carcinogenesis	CCL20	promote aggressive tumour behaviour	2016	[166]
EC	Fusobacterium, and Streptococcus	an increase in Fusobacterium and a decrease in Streptococcus	carcinogenesis	/	/	2019	[164]
EC	Streptococcus, Neisseria, Haemophilus, and Porphyromonas	an increase in Neisseria, Haemophilus, and Porphyromonas, and a decrease in Streptococcus	carcinogenesis	/	/	2020	[165]
EC	Porphyromonas gingivalis	increased	carcinogenesis	/	/	2016	[168]

The human microbiome constitutes a complex ecosystem of microorganisms inhabiting the body, comprising trillions of bacteria, viruses, fungi and other microbes [33–36]. The seminal work by the Human Microbiome Project (HMP) has elucidated the extensive diversity and functionality of the microbiome, emphasising its significance in maintaining health and contributing to disease pathogenesis [37, 38]. Particularly dense in the gastrointestinal tract, the microbiome plays a pivotal role in numerous physiological processes, including digestion, immune function, and even brain health and behaviour [39–41]. Its impact extends beyond infectious diseases, encompassing a spectrum of human disorders, from metabolic disorders, neurodegenerative diseases, allergies and cardiovascular conditions to various cancers [42–46]. Subsequent research has deepened our understanding of the microbiome’s role in modulating the immune system, with evidence suggesting that dysbiosis, an imbalance in microbial communities, can influence autoimmune disease development and infectious disease response [47–50].

**Fig. 1:**
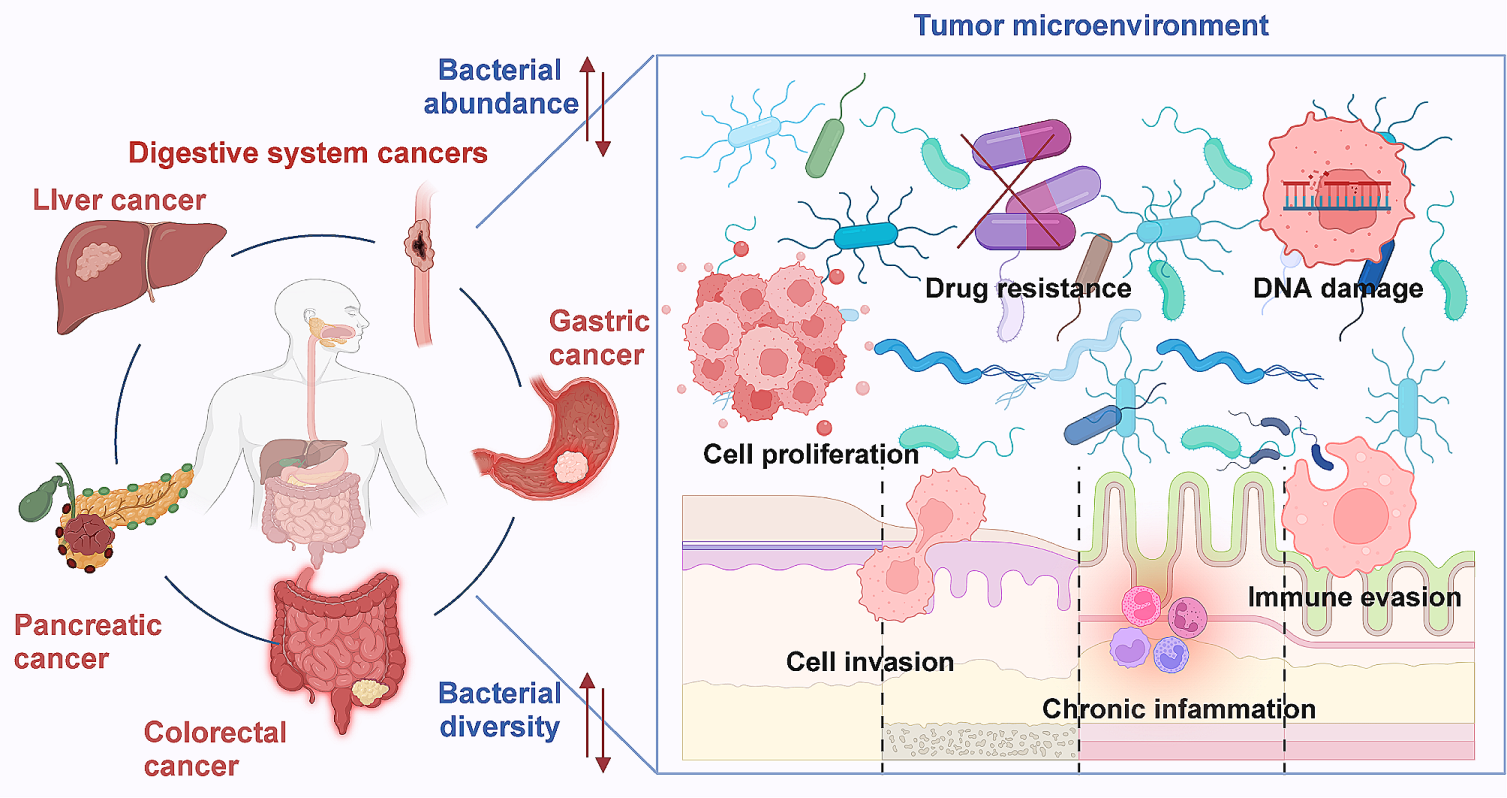
The pivotal roles of intratumoral bacteria in the pathogenesis of gastrointestinal cancers. Diverse gastrointestinal cancers exhibit significant alterations in the diversity and abundance of bacterial communities, with strong links to cancer progression. The enrichment and colonisation of specific bacterial populations within tumour tissues lead to significant reshaping of the tumour microenvironment. Through various direct and indirect mechanisms, these bacteria contribute to cancer development by promoting chronic inflammation, inducing DNA damage and enhancing cellular proliferation and invasion. Moreover, the bacteria associated with digestive system cancers exert a dual role in cancer dynamics, capable of both driving and impeding tumour progression. Their influence extends across multiple malignant processes, including immune system evasion, persistent inflammation stimulation and genetic instability promotion within host cells

**Fig. 2:**
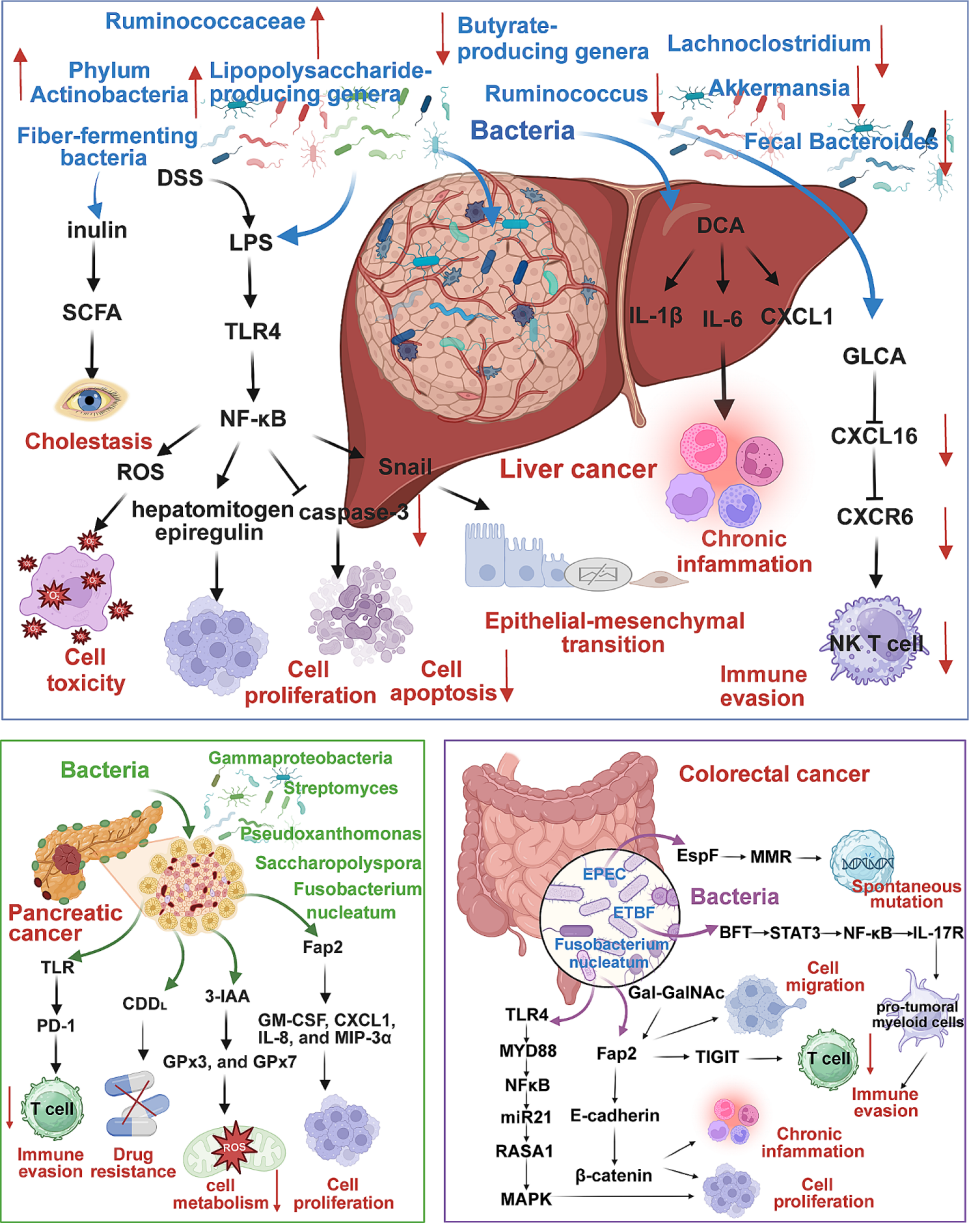
Major characteristic alterations and molecular mechanisms of intratumoral bacteria in digestive system cancers. In digestive system cancers, significant changes in the diversity and abundance of bacterial communities underscore an abnormal bacterial landscape, highlighting their significant roles in cancer development. These aberrant bacterial communities influence cancer progression through a myriad of mechanisms, engaging in both direct and indirect interactions. Directly, bacteria interact with cancer cells or modulate the tumour microenvironment, whereas indirectly, they exert effects through the secretion of virulence factors or the generation of metabolic byproducts. Such interactions significantly affect cancer-associated molecular pathways and cellular processes, thereby playing a critical role in the regulation of tumour progression. Therefore, these altered bacteria are implicated in various malignant processes. They contribute to the proliferation and invasion of tumour cells, facilitate the epithelial-mesenchymal transition (EMT) and regulate inflammation, apoptosis, immune evasion and bile acid metabolism. Furthermore, they influence oxidative stress and DNA damage, underscoring their comprehensive impact on the pathogenesis of digestive system cancers

Moreover, recent advancements in sequencing technologies and microbiome research have unveiled the complex interplay between intratumoral bacteria and cancer, prompting significant interest in their potential roles in oncogenesis and malignancy progression [51–55]. Emerging evidence indicates that intratumoral bacteria, situated within the tumour microenvironment, contribute to the modulation of carcinogenic processes through diverse mechanisms, including carcinogen production, host inflammation modulation and tumour microenvironment alteration [56–58]. The complex and dynamic nature of the bacterial ecosystem within the digestive system underscores the pivotal role of intratumoral bacteria in digestive system cancers (Fig. 1) [59]. These bacteria exhibit dual functions in digestive system cancers, serving as promoters of oncogenesis while also acting as protective agents against tumorigenesis, depending on their interactions with host cells and the local microenvironment (Fig. 2) [60–63]. For instance, the association between *Helicobacter pylori* (*H. pylori*) infection and gastric cancer (GC) exemplifies the significant impact of certain intratumoral bacteria on the onset of digestive system malignancies [64]. Conversely, certain bacterial populations within colorectal cancer (CRC) may beneficially modulate immune responses by producing short-chain fatty acids from dietary fibres, suggesting the potential of dietary interventions in CRC management [65–68]. Importantly. the influence of intratumoral bacteria extends beyond cancer initiation and progression to significantly affect patients’ responses to treatment and overall prognoses [69, 70]. Intratumoral bacteria play a role in modulating the tumour microenvironment, influencing drug metabolism, and interacting with the host immune system, which can either enhance or impair the efficacy of existing therapies. For instance, specific bacteria within CRC have been associated with improved immunotherapy efficacy, potentially due to the influence on the immune microenvironment, favouring T cell activation and infiltration [71]. This observation highlights the potential for microbiome profiling to predict response to immunotherapy and guide treatment decisions. Additionally, certain bacteria have been found to metabolise chemotherapeutic drugs, such as gemcitabine, reducing the drug’s availability and efficacy in pancreatic cancer treatment [72, 73]. This emphasises the importance of considering intratumoral microbiota in designing and administering chemotherapeutic regimens. As research progresses, manipulating intratumoral bacteria through antibiotics, probiotics, or dietary interventions is emerging as a novel strategy to enhance treatment efficacy in multiple digestive system cancers [71, 74–77]. Furthermore, the composition of intratumoral microbiomes correlates with patient prognosis, with specific microbial signatures associated with either improved or worsened survival outcomes [78, 79]. For example, reduced diversity of intratumoral bacteria in CRC has been associated with a better prognosis, possibly due to the beneficial effects of microbial diversity on immune system regulation [79]. Collectively, these findings underscore the profound impacts of intratumoral bacteria in digestive system cancers, highlighting the implications for integrating microbiome research into digestive system cancer treatment (Table 2) [80–83].

**Table 2:** The clinical significance of intratumoral bacteria in digestive system cancers

Cancer type	Bacteria and other pathogens	Role	Sample type	Clinical application	Publication year	Reference
HCC	a decrease in Escherichia coli and Shigella, and an increase in Faecalibacterium, Ruminococcus, Ruminoclostridium	carcinogenesis	486 faecal samples from East China, Central China, and Northwest China	diagnosis	2019	[61]
HCC	a decrease in Escherichia coli and Shigella, and an increase in Faecalibacterium, Ruminococcus, Ruminoclostridium	carcinogenesis	faeces from 33 healthy controls, 35 individuals with B-HCC and 22 individuals with NBNC-HCC	diagnosis	2019	[87]
HCC	a decrease in Escherichia coli and Shigella, and an increase in Faecalibacterium, Ruminococcus, Ruminoclostridium	carcinogenesis	faeces from patients with 21 NAFLD-related cirrhosis and HCC, 20 NAFLD-related cirrhosis without HCC, and 20 healthy controls	diagnosis	2019	[62]
GC	Bacterial diversity	carcinogenesis	tissue samples from 54 patients with GC and 81 patients with chronic gastritis	diagnosis	2018	[141]
GC	Bacterial diversity	carcinogenesis	tissue samples from 5 non-atrophic gastritis, 5 intestinal metaplasia, and 5 intestinal-type GC	diagnosis	2014	[140]
GC	Bacterial diversity	carcinogenesis	gastric mucosal samples from 81 cases including superficial gastritis, atrophic gastritis, intestinal metaplasia, and GC	diagnosis	2018	[139]
GC	Bacterial diversity	carcinogenesis	gastric wash samples from 6 GC and 5 superficial gastritis	diagnosis	2018	[142]
GC	Bacterial composition and diversity	carcinogenesis	tissue samples from 276 GC	diagnosis	2019	[145]
EC	Streptococcus, Neisseria, Haemophilus, and Porphyromonas	carcinogenesis	paired oesophageal biopsy and swab specimens from 236 patients with EC	diagnosis	2020	[165]
HCC	LPS	carcinogenesis	a splenic vein metastasis of the nude mouse model, HepG2, Huh7, Hep3B, SMMC-7721 and MHCC97-H HCC cell lines, and 106 clinical tissue samples from HCC patients	prognosis	2012	[78]
CRC	Fusobacterium nucleatum	carcinogenesis	HCT116 and LoVo cells, a xenograft animal model, 90 tumour and matched nontumor tissues from patients in China, and 125 tumour tissues from patients in Japan	prognosis	2017	[79]
GC	Fusobacterium and Prevotella	carcinogenesis	tissue samples from 64 GC and 64 non-tumorous GC	prognosis	2023	[146]
PC	Pseudoxanthomonas, Saccharopolyspora and Streptomyces	anti-carcinogenesis	human tissues from 36 long-term PC survivors and 32 PC patients who survive less than 5 years, and an orthotopic mouse PC model	prognosis	2019	[63]
PC	somatic-cell-associated bacteria	carcinogenesis	41 human PC tumour samples and 14 normal pancreatic tissues	prognosis, and immunotherapy	2022	[158]
EC	Fusobacterium nucleatum	carcinogenesis	325 resected EC specimens	prognosis	2016	[166]
EC	Porphyromonas gingivalis	carcinogenesis	oesophageal tissues from 100 EC patients and 30 normal controls	prognosis	2016	[168]
HCC	DCA	carcinogenesis	an obesity-associated HCC mouse model, and NASH-related HCC patients	targeted therapy	2013	[74]
HCC	GLCA	carcinogenesis	a primary liver mouse model, liver metastasis mouse models and 142 patients of the TIGER-LC Consortium	antitumor immunity	2018	[75]
HCC	SCFA	carcinogenesis	T5KO mouse models treated with a high-fat diet enriched with inulin	dietary interventions for preventing HCC	2018	[76]
CRC	Fusobacterium nucleatum	carcinogenesis	primary human NK cells, DCs, TILs, CRC RKO cells, mouse thymoma BW cells, and the NK tumour cell line YTS ECO	antitumor immunity	2015	[103]
CRC	ETBF	carcinogenesis	colonic epithelial cells, and a NETBF-colonised ApcMin mouse model	antitumor immunity	2018	[71]
PC	Bacterial composition and diversity	carcinogenesis	human faecal samples from healthy volunteers and PC patients, preinvasive and invasive PC mouse models	immunotherapy	2018	[77]
PC	Gammaproteobacteria	carcinogenesis	113 human PC tissue samples, and 20 normal human pancreas samples	chemotherapy	2017	[72]
PC	3-IAA	anti-carcinogenesis	23 human PC tissues from 11 therapy responders and 12 therapy non-responders and PC mouse models	chemotherapy	2023	[157]

**Fig. 3:**
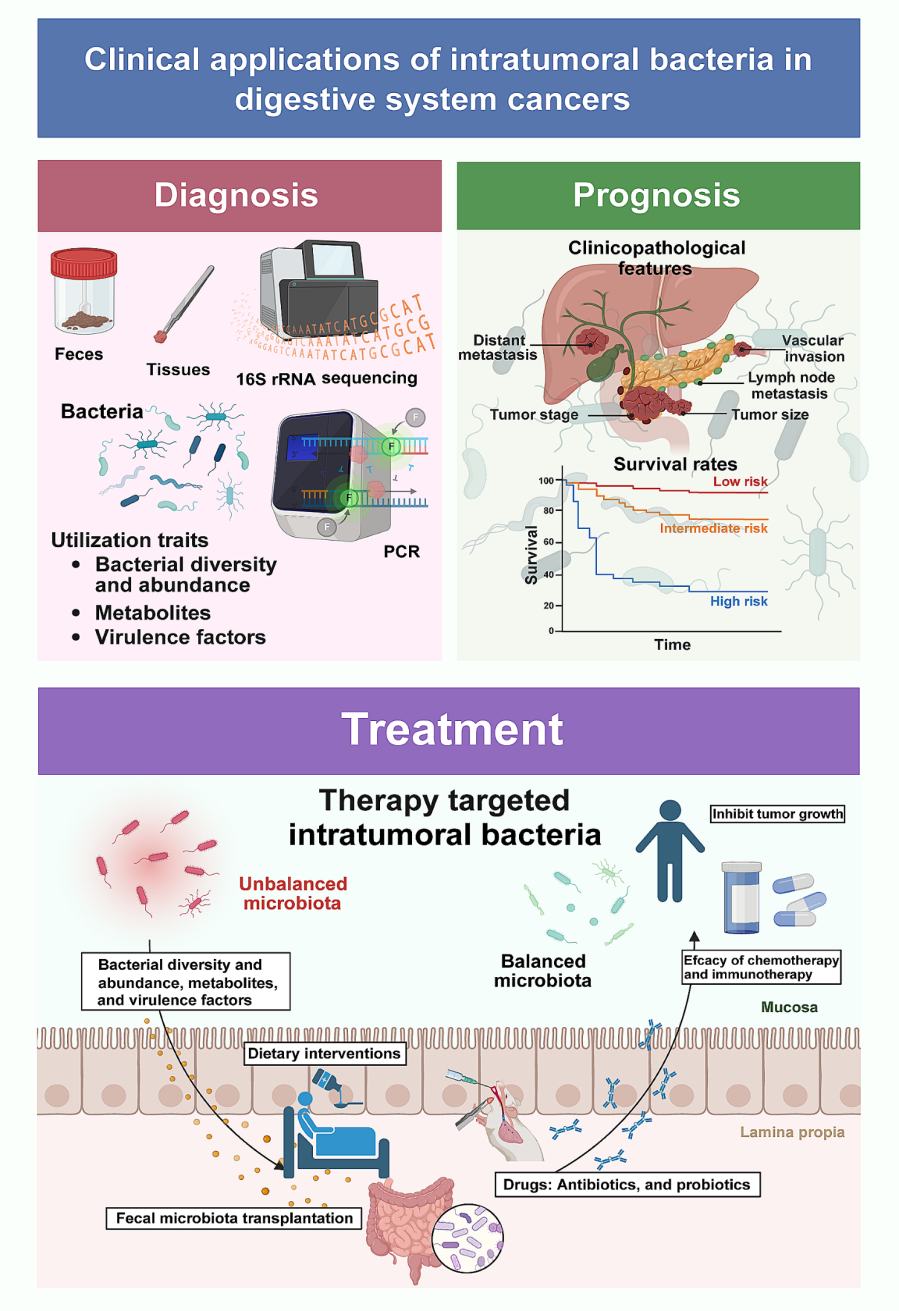
Clinical potential of intratumoral bacteria in digestive system cancers The unique bacterial signatures correlated with various stages and types of digestive system cancers offer novel avenues for utilising certain bacterial species as non-invasive biomarkers, with promise potential as early detection and accurate diagnostic markers of digestive system cancers. In terms of prognosis, the composition of bacterial communities within tumours is intricately linked to a range of clinicopathological features and can profoundly influence survival outcomes. Therapeutically, innovative approaches targeting these bacterial populations and their metabolic outputs have shown promising results. The strategic application of antibiotics, probiotics and faecal microbiota transplantation has been found to significantly inhibit tumour growth. Furthermore, these microbial-based therapies enhance the sensitivity of tumours to conventional chemotherapies and immunotherapies

This review provides a comprehensive analysis of intratumoral bacteria in digestive system cancers, with a specific focus on liver cancer (LC), CRC, GC, pancreatic cancer (PC) and oesophageal cancer (EC). Our objective is to consolidate the diversity of bacteria found within digestive system cancers, elucidate their proposed mechanisms, and discuss implications for digestive system cancer therapy. By exploring the intersection of microbiology and digestive system cancers, this review illuminates the potential of intratumoral bacteria as biomarkers for diagnosis and prognosis, as well as potential targets for innovative therapeutic strategies in digestive system cancers (Fig. 3).

## Roles and mechanisms of intratumoral bacteria in digestive system cancers

### Roles and mechanisms of bacteria in LC

The complex relationship between gut microbiota and the development of LC is increasingly evident, influenced by both local and systemic factors. The liver, which receives nutrient-rich blood from the intestine via the portal vein, is a key site for interactions with the gut microbiome and microbe-associated molecular patterns (MAMPs). These MAMPs typically consist of microbial metabolites and byproducts [84]. Extensive research has highlighted the critical role of intestinal permeability and dysbiosis in facilitating the progression of chronic liver diseases to hepatocellular carcinoma (HCC), highlighting bidirectional regulation and interconnection between these factors [85, 86].

Recent studies have identified distinct variations in microbial diversity between patients with liver cirrhosis and those with HCC. Notably, the progression from liver cirrhosis induced by chronic hepatitis B virus (HBV) infection to early-stage HCC is marked by an increase in fecal microbial diversity. This includes a rise in the Phylum Actinobacteria and lipopolysaccharide-producing genera, coupled with a decrease in butyrate-producing genera. A random forest model utilising microbiota with differential expression, validated across 486 faecal samples from diverse regions, achieved an 80.64% area under the curve for early HCC diagnosis, indicating the potential of microbial profiles in non-invasive diagnostics [61]. Additionally, faecal microbial richness is significantly higher in patients with HBV-related HCC compared to healthy controls and patients with non-HBV non-hepatitis C virus (NBNC)-related HCC. Faecal samples from patients with NBNC-related HCC exhibit a higher abundance of *Escherichia coli* and Shigella and lower concentrations of faecal *Bacteroides, Ruminococcus* and *Lachnoclostridium* [87]. Compared to NASH-induced liver cirrhosis without HCC, patients with HCC showed elevated levels of *Bacteroides* and Ruminococcaceae and decreased levels of *Akkermansia* [62]. The variable features of specific microbial profiles in patients with HCC offer a promising non-invasive approach based on microbial-based diagnostic tests. Moreover, elevated levels of lipopolysaccharides (LPS), a cell wall component of gram-negative bacteria, have been detected in various HCC cell lines, mouse models and blood samples, confirming the phenomenon of intestinal leakage across different stages of chronic liver disease progression to HCC [88]. Increased intestinal permeability induced by agents like dextran sodium sulfate elevates portal LPS levels and promotes tumour formation in a choline-deficient high-fat diet-induced non-alcoholic steatohepatitis (NASH) mouse model [89]. Furthermore, enhanced intestinal bacterial translocation contributes to chronic liver inflammation through the interaction of MAMPs with pattern recognition receptors (PRRs) on host liver-resident cells (including hepatocytes, human hepatic stellate cells and Kupffer cells), thereby facilitating liver cancer development [88]. Increased LPS levels stimulate both human and mouse hepatic stellate cells to upregulate epiregulin mRNA and protein in a nuclear factor kappa B (NF-κB)-dependent manner, exerting a potent mitogenic effect on hepatocytes [90]. Additionally, activation of the LPS-TLR4 axis results in reduced cleavage of the apoptotic marker caspase-3 mediated by NF-κB, thereby preventing hepatocyte apoptosis [90]. Furthermore, sustained activation of the LPS-TLR4 axis promotes NF-κB activation and weakens reactive oxygen species-induced toxicity, amplifying inflammation-mediated hepatocyte proliferation and tumorigenic response [88]. Additionally, LPS-induced constitutive overexpression of TLR4 directly activates NF-κB signalling in HCC SMMC-772 cells, leading to the activation of the main transcription factors involved in epithelial-mesenchymal transition (EMT) such as Snail [78]. Consequently, Snail induction in SMMC-7721 cells enhances EMT processes and invasion in HCC SMMC-7721 cells. Notably, TLR4 is expressed in the majority of clinical tissue specimens from 106 patients with HCC, and its high expression carries worse prognostic implications, correlating with unfavourable cancer-free or overall survival. TLR4 overexpression is closely associated with poor clinicopathologic characteristics, including cirrhosis, tumour size, margin, vascular invasion, UICC T stage, portal vein thrombosis and tumour thrombus.

Furthermore, dysbiosis has been implicated in mediating liver cancer development through bacterial metabolites. Studies in obesity-associated HCC mouse models have revealed alterations in gut microbiota and increased levels of deoxycholic acid (DCA) levels, promoting the transformation of hepatic stellate cells into a senescence-associated secretory phenotype (SASP). This phenotype releases inflammatory and tumour-promoting factors such as IL-6, CXCL1 and CXCL9. Interestingly, vancomycin treatment, which targets Gram-positive bacteria, attenuated signs of SASP and slowed HCC progression, suggesting that the increase in obesity-related Gram-positive bacteria may facilitate HCC development via the enterohepatic circulation of gut bacterial metabolites. Importantly, the phenomenon of cellular senescence and SASP has also been observed in the HSCs of NASH-related patients with HCC. Moreover, gut bacteria-controlled bile acid metabolism has been implicated in liver antitumor immunosurveillance. The role of Gram-positive bacteria in primary-to-secondary bile acid transformation reduces chenodeoxycholic acid (CDCA) concentration and increases glycolithocholate (GLCA), thereby decreasing CXCL16 expression on liver sinusoidal endothelial cells. This imbalance suppresses CXCR6 + Natural Killer T (NKT) cell accumulation and antitumor activity, thus promoting liver tumour growth [75]. Similar regulatory effects of bile acids CXCL16 expression have been confirmed in human liver sinusoidal endothelial cells and tissue samples. Additionally, dietary soluble fibres and their fermentation products, short-chain fatty acids (SCFA), exhibit complex effects during icteric HCC development. Toll-like receptor 5 deficient (T5KO) mouse models, prone to icteric HCC, demonstrate gut dysbiosis characterised by an increase in fibre-fermenting bacteria and *Proteobacteria*. A high-fat diet supplemented with inulin in T5KO mouse models leads to gut dysbiosis, early onset of cholestasis, hepatocyte death, neutrophilic inflammation and eventual formation of icteric HCC. Targeted interventions aimed at reducing fibre-fermenting bacteria, inhibiting fermentation, lowering soluble fiber intake, or preventing bile acid reabsorption have significantly reduced HCC incidence. These findings underscore the gut-microbiota-liver axis as a critical target for HCC treatment strategies [76].

Targeting the gut-microbiota-liver axis, with interventions directed at LPS and its receptor TLR4, has shown promise in mitigating HCC development across preclinical studies [91]. Continuous gut decontamination with antibiotics such as ampicillin, neomycin, metronidazole and vancomycin effectively reduces tumour number and size in HCC mouse models. Furthermore, the modulation of the gut microbiome influences responses to chemotherapy and immunomodulatory therapy [84, 92]. Interventions including faecal microbiota transplantation (FMT), TLR antagonists and the use of bile acids and their receptors to protect the intestinal barrier have demonstrated therapeutic potential in various experimental mouse models [85]. However, the translation of these preclinical findings from mice and rats into clinical trials remains a gap, underscoring the therapeutic potential of targeting the gut-microbiota-liver axis in future HCC treatment strategies.

## Roles and mechanisms of bacteria in CRC

Increasing large-scale metagenomic studies in human CRC have underscored species-specific microbial compositional and ecological alterations, highlighting the pivotal role of the intestinal microbiota in the tumorigenesis of CRC [68, 93–95].

A strong association between gut microbial dysbiosis and CRC pathogenesis implicates the involvement of the gut microbiome in CRC initiation and progression through diverse complex mechanisms [96]. For instance, Fusobacteria, commonly found in the human oral cavity, has been detected in higher abundance in CRC tissues compared to healthy controls, suggesting its role in promoting tumour growth and CRC progression [97–100]. Recent research indicates that the host factor D-galactose-β (1–3)-N-acetyl-D-galactosamine (Gal-GalNAc) is overexpressed in CRC, facilitating *Fusobacterium* enrichment in CRC by binding with the galactose-binding lectin, Fap2, on *Fusobacterium nucleatum*’s surface. This interaction promotes CRC metastasis, emphasising the critical role of host-microbe interactions in CRC progression [101]. Additionally, *Fusobacterium nucleatum* activates β-catenin signalling by binding its FadA adhesin to E-cadherin on CRC cells, promoting invasion, inflammatory responses and CRC cell proliferation, thereby contributing to CRC oncogenesis [102]. Mechanistically, *Fusobacterium nucleatum* stimulates the NFκB pathway through the activation of the TLR4/MYD88 cascade in HCT116 cells, leading to the upregulation of downstream target miR21 expression. Subsequently, *Fusobacterium nucleatum* manipulates miR21-mediated RAS p21 protein activator 1 (RASA1) downregulation to activate the MAPK pathway, enhancing invasiveness and proliferation of CRC cells. Importantly, higher levels of *Fusobacterium nucleatum* and miR21 are associated with advanced CRC phenotypes, including late T stage, elevated Ki-67 expression and lymphatic invasion, leading to reduced overall survival rates in patients with CRC [79]. These findings suggest a close association between *Fusobacterium nucleatum* abundance and adverse clinical outcomes in CRC, with implications for prognosis prediction and clinical management.

Furthermore, *Fusobacterium nucleatum* develops evasion mechanisms by inhibiting the infiltration and cytotoxic activity of Natural Killer (NK) cells within CRC tumours. The interaction between the Fap2 protein of *Fusobacterium nucleatum* and the inhibitory receptor T cell immunoglobulin and ITIM domain (TIGIT) on human NK cells hampers the activity of NK cells, protecting human colon tumours from immune cell attack [103]. Similarly, studies have demonstrated the causative roles of *Bacteroides fragilis* in CRC [104, 105]. Enteropathogenic *Escherichia coli* (EPEC) infection induces the translocation of the EPEC-secreted effector protein EspF to the host cell membrane and mitochondria, depleting DNA mismatch repair (MMR) proteins in host HT29 and SW480 cells and increasing the spontaneous mutation frequency, contributing to CRC development [106]. Additionally, Enterotoxigenic *Bacteroides fragilis* (ETBF) secrets Bacteroides fragilis toxin (BFT) to activate colonic epithelial cells via STAT3/NF-κB/IL-17R signalling, leading to Th17 cell response and CXCR2-expressing polymorphonuclear immature myeloid cell recruitment and consequently inducing a pro-carcinogenic inflammatory response and myeloid-cell-dependent distal colon tumorigenesis [71]. These findings demonstrate that bacteria can disrupt the tumour’s immune microenvironment, facilitating CRC formation.

### Roles and mechanisms of bacteria in GC

GC presents a significant global health challenge, with *H. pylori* infection identified as the most unequivocal risk factor, implicated in nearly all GC cases and instigating pathogenic mechanisms crucial to disease progression [107–114]. These mechanisms include initiating a chronic inflammatory response, manipulation of the host’s innate immune system, inducing DNA damage and dysregulating autophagy pathways [115–119]. *H. pylori* colonises the gastric mucosa of over half of the global population, yet only a subset develops GC, suggesting a complex interplay of bacterial, host and environmental factors. For example, *H. pylori* infection initiates a persistent inflammatory response in the gastric mucosa, characterised by the recruitment of inflammatory cells and the release of pro-inflammatory cytokines such as IL-1β, IL-8 and TNF-α. This inflammatory milieu fosters a conducive environment for neoplastic transformation, seen as a precursor to atrophic gastritis, a well-established GC risk factor [120–122]. Moreover, *H. pylori* secret virulence factors, notably cytotoxin-associated gene A (CagA) and vacuolating cytotoxin A (VacA), that manipulate host cell proliferation and apoptosis [123–125]. CagA, upon injection into host cells, undergoes phosphorylation and interacts with multiple signal transduction pathways, leading to dysregulated cell signalling, proliferation and increased mutations [126–129]. *H. pylori* infection is also associated with increased DNA damage within gastric epithelial cells due to the production of reactive oxygen and nitrogen species inducing oxidative stress, resulting in genomic instability in key genes involved in gastric carcinogenesis [130–133]. Beyond *H. pylori*, advancements in polymerase chain reaction (PCR) technology and metagenomics have revealed a complex gastric microbiota potentially contributing to GC development [134–137]. Studies also explore *H. pylori*’s role in modulating the gastric microbiome, suggesting *H. pylori*-induced dysbiosis may further contribute to carcinogenesis [138]. Consistently, reduced gastric microbial diversity is observed from atrophic gastritis (AG) to intestinal metaplasia (IM), and finally to GC, suggesting a dysbiotic shift favouring oncogenic processes [139–142]. Conversely, some findings show increased microbial evenness and diversity in patients with GC compared to those with other gastritis forms, indicating a complex relationship between microbial diversity and cancer development [143, 144]. Recent research focusing on microbial profiling from healthy gastric mucosa to GC in individuals has identified a significant decrease in microbial diversity and richness within peritumoral and tumoral microhabitats [145]. Moreover, the combination of specific dysregulated bacterial clusters has been identified with a strong discriminatory capability for distinguishing between gastritis and GC, highlighting their potential as biomarkers for early detection. Additionally, variations in gastric microbiota among patients with GC have prognostic significance, with certain bacteria like *Fusobacterium* and *Prevotella* significantly associated with poorer overall survival rates, suggesting their potential as prognostic targets [146]. Investigation into the gastric microbiome’s role in GC underscores the need for a comprehensive understanding of cancer development, considering not only pathogenic bacteria like *H. pylori* but also the broader microbial ecosystem. This approach holds promise for innovative diagnostic, prognostic and therapeutic strategies, potentially revolutionising GC management through targeted modulation of the gastric microbiota [136, 147–149].

### Roles and mechanisms of bacteria in PC

PC, notorious for its dismal prognosis and rapid progression, has recently been associated with a distinct intratumoral microbiome [150–153]. Studies indicate that bacteria residing within the PC tumour microenvironment significantly contribute to cancer development, immune evasion and treatment resistance [154–156]. Pushalkar et al. pioneered the discovery of a unique bacterial population within pancreatic tumours, challenging the previous notion of the pancreas as a sterile environment [77]. Their research revealed a substantially richer microbiome in cancerous pancreas compared to normal pancreatic tissue in both mice and humans. This microbiome plays a crucial role in shaping the tumour’s immune landscape to foster immune tolerance. The enriched microbial ecosystem activates TLR on macrophages, leading to an increase in myeloid-derived suppressor cells (MDSCs), suppression of Th1 polarisation of CD4 + T cells and activation of CD8 + T cells. These changes diminish the effectiveness of immune checkpoint therapies by inhibiting PD-1 expression. Depleting the tumour-associated microbiome with oral antibiotics has shown promise in reversing this microbiome-induced immune tolerance, reducing tumour burden by approximately 50% and enhancing the efficacy of PD-1-based immunotherapies [77]. These findings underscore the complex interplay between the tumour microbiome and the host immune system in PC, highlighting the potential of microbiome-targeted therapies as adjuncts to immunotherapy. Previous research also highlights the intricate impact of intratumoral bacteria on chemotherapy efficacy. Bacterial species within PC, particularly Gammaproteobacteria, metabolise gemcitabine, a standard PC chemotherapy drug, into its inactive form by expressing a specific form of the bacterial enzyme cytidine deaminase (CDDL), contributing to chemoresistance [72]. Additionally, recent studies elucidate a complex interplay between microbiome-derived metabolites and the innate immune response within the tumour microenvironment, ultimately influencing chemotherapy efficacy in PC. A significant correlation was observed between the levels of the microbiota-derived tryptophan metabolite indole-3-acetic acid (3-IAA) and treatment outcomes in two independent PC cohorts, with higher concentrations of 3-IAA enriched in patients who responded to chemotherapy [157]. Neutrophil-derived myeloperoxidase plays a pivotal role in this context by oxidising 3-IAA. Combined with chemotherapy, this oxidation process induces the downregulation of reactive oxygen species (ROS)-degrading enzymes, specifically glutathione peroxidase 3 (GPx3) and GPx7, impairing cancer cell metabolic adaptability and hindering their proliferative capacity. While some bacteria contribute to immune evasion and therapy resistance, others can enhance immune surveillance and suppression of tumour growth [154]. Beneficial microbiota also exists within PC tissues. 16 S rRNA gene sequencing analysis was conducted using two independent cohorts of patients with PC who survived for more than five years (long-term survivors, LTS) and those who did not survive for five years. The results revealed a higher microbial diversity in LTS tissues. A univariate Cox proportional hazards model further demonstrated that patients with higher microbial diversity within PC exhibited significantly prolonged overall survival. A combination of microbial genera consisting of *Pseudoxanthomonas*, *Saccharopolyspora* and *Streptomyces* within the tumour microbiome has been identified as a strong predictor of favourable long-term survival outcomes in patients with PC, thereby offering new avenues for PC therapeutic interventions. Human-to-mouse FMT experiments also reveal that modulating these three bacterial genera within PC tumours enhances CD8 + T cell recruitment and immune response, inhibiting tumour growth. This mechanism suggests a direct link between specific components of the tumour microbiome and the activation of effective antitumor immunity [63]. Additionally, tumour-microbiome crosstalk is increasingly recognised as pivotal in regulating PC tumorigenesis. Single-cell analysis of host-microbiome interactions (SAHMI), drawing on data from two large, independent scRNA-seq cohorts of pancreatic ductal adenocarcinoma (PDA), has highlighted the significant role of somatic-cell-associated bacteria in association with tumour cells, influencing cell motility and immune signalling pathways [158]. Furthermore, the presence of cell-associated bacteria within PC across multiple independent datasets emphasises their potential as valuable prognostic markers. High intratumoral levels of *Fusobacterium nucleatum* in PC have also been linked to shorter survival in patients. *Fusobacterium nucleatum* infection induces both normal pancreatic epithelial cells and PC cells, driven by Fap2, to secrete elevated levels of cytokines such as granulocyte-macrophage colony-stimulating factor (GM-CSF), chemokine (C-X-C motif) ligand 1 (CXCL1), Interleukin-8 (IL-8) and macrophage inflammatory protein-3α (MIP-3α). These cytokines, through host autocrine and paracrine signalling, enhance phenotypes in PC BxPC3 and Panc1 cells associated with tumour progression, including cell proliferation, migration and invasion, thus contributing to the aggressive nature of PC [159].

The findings underscore highlights the potential of microbiome-targeted therapies as complementary to conventional cancer treatments. By selectively promoting the growth of beneficial bacteria or reducing harmful microbial populations, it may be possible to improve patient outcomes in PC.

### Roles and mechanisms of bacteria in EC

Recent investigations into the oesophageal microbiome have revealed significant alterations in patients with EC, shedding light on the potential influence of the microbiome on the development of this malignancy [160–162]. Comparisons between healthy individuals, patients with Barrett’s oesophagus (BE) and those diagnosed with EC have shown clear distinct microbial compositions, highlighting the dynamic changes accompanying disease progression. Particularly noteworthy is the observed decrease in microbial diversity among patients with EC, characterised by a shift from the abundance of *Veillonella* and *Streptococcus* to the predominance of *Lactobacillus* [163]. This shift is believed to significantly impact the tumour microenvironment, potentially contributing to tumorigenesis. Further analysis of microbial diversity in EC tissues, conducted using 16 S rDNA sequencing, has shown a significant decrease compared to nontumor tissues, accompanied by an increase in *Fusobacterium* abundance and a corresponding decrease in *Streptococcus* [164]. Innovative research utilising microbial prediction models has identified the combination of *Streptococcus* and *Neisseria* as effective predictors of EC progression and its precancerous lesions, demonstrating high diagnostic performance, with an area under the curve (AUC) value of 0.738, and potential for early detection and progression monitoring [165]. Subsequent studies involving 325 resected EC specimens have revealed significantly higher levels of *Fusobacterium nucleatum* DNA in EC tissues compared to normal oesophageal mucosa. The presence of *Fusobacterium nucleatum* DNA strongly correlated with advanced tumour stages and shorter cancer-specific survival, indicating its potential as a prognostic biomarker in EC [166]. Additionally, a significant correlation has been observed between *Fusobacterium nucleatum* and the chemokine CCL20, suggesting a potential mechanism through which *Fusobacterium nucleatum* may promote tumour aggressiveness [166]. *Porphyromonas gingivalis* has also been identified as more prevalent in EC tissues, with its abundance varying across different stages of EC [167, 168]. Its presence is often associated with poor tumour differentiation, advanced stages, metastasis and reduced survival outcomes, further emphasising its prognostic significance. Emerging evidence supports the role of the oesophageal microbiome in modulating responses to immune checkpoint inhibitors in EC, with different microbial compositions correlating with varying responses to radiotherapy and chemotherapy [161, 169, 170]. These insights underscore the intricate relationship between intratumoral bacteria and the host immune system in EC, highlighting the potential of microbiome-targeted therapies to complement traditional cancer treatments and improve therapeutic efficacy and patient outcomes [171, 172].

## Conclusion

The intricate interactions of bacteria within digestive system cancers present a rapidly expanding area of research at the intersection of microbiology and oncology. Intratumoral bacteria contribute to digestive system cancer pathogenesis through direct and indirect interactions, influencing cancer cell proliferation, genetic and epigenetic alterations, and the tumour microenvironment conducive to tumour growth and metastasis. This review provides a comprehensive summary of the current understanding of how intratumoral bacteria shape the pathogenesis, diagnosis and treatment of digestive system cancers. By examining recent studies, we elucidate the distinct microbial profiles associated with various digestive system cancers and their roles in inflammation promotion, immune response modulation and tumour microenvironment alteration. We further explore the diagnostic and prognostic potential of microbial signatures in digestive system tumours, indicating their value in predicting disease progression and treatment outcomes. Specific bacterial signatures within tumours have been linked to disease stage, treatment responses and patient survival, suggesting potential applications in precision medicine. Nevertheless, capitalizing on the therapeutic possibilities of targeting these microbial communities poses significant challenges, necessitating a delicate balance between disrupting pathogenic interactions and preserving beneficial host-microbiome relationships.

This review underscores several key limitations in the current research, primarily the associative rather than causative understanding of intratumoral bacteria’s roles in digestive system cancers, and the microbiome’s dynamic responses to disease progression and treatment. Future studies should focus on elucidating the mechanistic foundations of how intratumoral bacteria influence the pathogenesis and progression of digestive system cancers. Advanced models enabling real-time study of tumour-microbiome interactions in situ will be essential. Moreover, exploring the therapeutic potential of modulating the tumour microbiome through targeted antibiotics, probiotic supplementation or FMT holds promise for improving cancer treatment outcomes.

In summary, investigating intratumoral bacteria in digestive system cancers offers new insights into tumour biology, with significant implications for diagnosis, prognostication and therapy. As the field progresses, employing multidisciplinary approaches that integrate microbiology, oncology and immunology will be crucial in fully harnessing the potential of microbiome-targeted therapies in cancer management.

### Electronic supplementary material

Below is the link to the electronic supplementary material.

Supplementary Material 1

Supplementary Material 2

## Data Availability

Not applicable.

## Abbreviations

MDSCs: myeloid-derived suppressor cells
HMP: Human Microbiome Project
H: pylori Helicobacter pylori
GC: gastric cancer
CRC: colorectal cancer
LC: liver cancer
HCC: hepatocellular carcinoma
PC: pancreatic cancer
EC: oesophageal cancer
MAMPs: microbe-associated molecular patterns
HBV: hepatitis B virus
NBNC: non-HBV non-hepatitis C virus
LPS: lipopolysaccharides
PRRs: pattern recognition receptors
NF-κB: nuclear factor kappa B
EMT: epithelial-mesenchymal transition
DCA: deoxycholic acid
SASP: senescence-associated secretory phenotype
CDCA: chenodeoxycholic acid
GLCA: glycolithocholate
NKT: Natural Killer T
SCFA: short-chain fatty acids
T5KO: Toll-like receptor 5 deficient
FMT: faecal microbiota transplantation
Gal-GalNAc: D-galactose-β (1–3)-N-acetyl-D-galactosamine
RASA1: RAS p21 protein activator 1
TIGIT T: cell immunoglobulin and ITIM domain
EPEC: Enteropathogenic Escherichia coli
MMR: mismatch repair
ETBF: Enterotoxigenic Bacteroides fragilis
