# Supplementary material for: Unravelling the role of intratumoral bacteria in digestive system cancers: current insights and future perspectives
Source: J Transl Med. 2024 Jun 7;22:545. doi: 10.1186/s12967-024-05320-6 (PMC11157735; doi:10.1186/s12967-024-05320-6)
Supplement: Supplementary file 2 — Supplementary Material 2 [file 12967_2024_5320_MOESM2_ESM.docx]

Dear Reviewer,

First and foremost, we would like to express our heartfelt thanks for your constructive comments and insights regarding the correct nomenclature for bacterial naming in our manuscript. We have carefully reviewed and adhered to the guidelines you provided, and we have made the necessary revisions to all previously incorrect bacterial nomenclature throughout the document.

These corrections have been highlighted in red in the revised manuscript to ensure they are easily identifiable. We believe that these changes have significantly enhanced the accuracy and rigor of our paper, aligning it with the high standards required for publication.

We hope that these amendments meet your expectations and address the concerns you raised in your review. Thank you once again for your meticulous review and valuable feedback, which have undoubtedly improved our work.

We look forward to your response and are eager to proceed with the publication process.

Warm regards,

Sincerely,

Huihui Gao
