# Supplementary material for: Unravelling the role of intratumoral bacteria in digestive system cancers: current insights and future perspectives
Source: J Transl Med. 2024 Jun 7;22:545. doi: 10.1186/s12967-024-05320-6 (PMC11157735; doi:10.1186/s12967-024-05320-6)
Supplement: Supplementary file 1 — Supplementary Material 1 [file 12967_2024_5320_MOESM1_ESM.docx]

Dear Editor，

We would like to express our heartfelt thanks to you and reviewers for your diligent and thorough review of our manuscript. Your insightful comments have been instrumental in enhancing the quality and clarity of our manuscript, ensuring that it meets the high standards of Journal of Translational Medicine.

We have carefully considered each point raised in the review and have made corresponding revisions to both the text and figures of our manuscript. We have also prepared a point-by-point response to each comment, which is included in this document, to illustrate how we have addressed your suggestions. We believe that the amendments made have significantly strengthened our manuscript, making it a more valuable contribution to the field.

Please feel free to contact us should you have any further questions or require additional information. We are more than willing to make further modifications if needed and look forward to your continued guidance.

Thank you once again for your invaluable support and constructive critiques.

Sincerely,

Huihui Gao

Reviewer reports:

Reviewer #1: The review is well-written and addresses multiple aspects of the microbiota-cancer relationship. I just have the following minor comments:

Second half of the sentence on page 6 "For example, reduced diversity of intratumoral bacteria in CRC has been associated with a better prognosis, possibly due to the beneficial effects of microbial diversity on immune system regulation [79]" seems to conflict with the first half regarding the value of "diversity". This conflict makes the statement on p7 "The transition from chronic hepatitis B virus (HBV) infection induced liver cirrhosis to early-stage HCC is characterised by an increase in faecal microbial diversity" unclear.

Response: Thank you for your thoughtful comments and the opportunity to clarify the seemingly conflicting statements regarding microbial diversity in digestive system cancers.

In the review, we aimed to illustrate that the dualistic role of microbial diversity within digestive system cancers, exhibiting both carcinogenic and anticarcinogenic effects depending on the type of cancer and the stage of its development. In colorectal cancer (CRC), for instance, specific bacterial populations within the tumor can enhance the efficacy of immunotherapy. Conversely, other studies have reported that an increase in microbial species within CRC is closely associated with poorer patient prognosis. This indicates that while microbial diversity can sometimes bolster immune system regulation, it may also contribute to adverse outcomes in certain contexts. In the case of the transition from chronic hepatitis B virus (HBV) infection-induced liver cirrhosis to early-stage hepatocellular carcinoma (HCC), an increase in fecal microbial diversity is observed. This increase underscores the complex impact that intratumoral bacteria have on the pathogenesis of digestive system cancers, highlighting the necessity of integrating microbiome research into cancer treatment strategies to fully understand and leverage these relationships.

These findings emphasize the intricate and context-dependent effects of microbial diversity within digestive system cancers, which could have significant implications for diagnosis, prognosis, and therapy in digestive system cancers.

The left panel of Fig 1 may be interpreted as a cycle, i.e. sequential events from GC to CR to PC to LC. Did you mean that?

Response: Thank you for your insightful observation regarding the left panel of Figure 1. The arrangement in the figure was not intended to suggest a sequential progression from GC to CR to PC to LC. Instead, the intent was to visually group these tumors as part of the digestive system cancers category.

To eliminate this ambiguity, we have already revised the figure by removing the arrows that might suggest a sequence. This modification ensures that the diagram accurately represents the tumors as distinct entities within the spectrum of digestive system cancers without implying any progression among them.

The manuscript already lists a plethora of references, but the following two recent references may add value:

Response: Thank you for your constructive suggestions regarding the additional references. We appreciate your efforts to enhance the comprehensiveness of our manuscript. Upon reviewing the two articles you recommended, we found them highly relevant and valuable for enriching the context and depth of our discussion. Accordingly, we have carefully incorporated these references into the appropriate sections of our manuscript. These additions help to bolster the evidence presented and broaden the perspective of our analysis.

Reviewer #3: I appreciate the authors' efforts in comprehensively reviewing and establishing the links between tomour-microbiome and digestive cancers. However, there are certain recommendations before the article could be considered for plausible publication:

1. Writing scientific names of microbial species should follow rules of scientific writing.

Response: Thank you for your valuable guidance regarding the correct formatting of microbial species names according to scientific writing standards. We acknowledge the importance of adhering to these conventions to ensure clarity and professionalism in our manuscript. In response to your suggestion, we have carefully reviewed our manuscript and corrected the nomenclature of all microbial species mentioned throughout the text. We have ensured that each name is now presented in the proper format, enhancing the scientific accuracy and readability of our work.

2. All the abbreviations should be abbreviated at the first instance even though the list of abbreviation is provided. for e. EC, PC, etc.

Response: Thank you for pointing out the necessity of defining abbreviations at their first instance in the manuscript. In accordance with your suggestion, we have meticulously revised the manuscript to ensure that all abbreviations, such as EC, PC, etc., are clearly defined upon their first use. This amendment helps to prevent any potential confusion and enhances the manuscript’s readability.

3. Excessive use of more general statements without references linking the two topics should be avoided

Response: Thank you for your recommendation to minimize the use of general statements without appropriate references that link the discussed topics. In response to your feedback, we have thoroughly reviewed the manuscript and revised any sections where the connections between topics were previously supported by overly general statements.

4. The para before conclusion, line 23'....aggressiveness', lacks reference.

Response: Thank you for your careful review and for pointing out the missing reference in our manuscript. We have revisited the section mentioned and have added the appropriate citation to support the statement concerning '‘aggressiveness.'

5. The language could be improved further.

Response: Thank you for your feedback regarding the language used in our manuscript. In response to your suggestion, we have engaged a professional language editor to revise our manuscript, enhancing its clarity, precision, and readability. We are confident that these improvements have significantly elevated the quality of our manuscript.
